# A preliminary multicenter evaluation of endoscopic sublay repair for ventral hernia from China

**DOI:** 10.1186/s12893-020-00888-4

**Published:** 2020-10-12

**Authors:** Rui Tang, Huiyong Jiang, Weidong Wu, Tao Wang, Xiangzhen Meng, Guozhong Liu, Xiaoyan Cai, Jianwen Liu, Xijun Cui, Xianke Si, Nan Liu, Nina Wei

**Affiliations:** 1 Department of Hernia and Abdominal Wall Surgery, East hospital affiliated to Tongji University, 150 Jimo Rd, Shanghai, 200120 China; 2 Department Two of General Surgery, Northeast International Hospital, Shenyang, Liaoning Province China; 3 Department of Gastrointestinal Surgery, Shanghai General Hospital, 86 Wujin Rd, Shanghai, China; 4 Department of Gastrointestinal Surgery, Linzi District Central Hospital, Zibo, Shandong Province China; 5 Department of General Surgery, Shengjing Hospital affiliated China Medical University, Shenyang, Liaoning Province China; 6 Department of Hepatopancreatobiliary and hernia Surgery, The First Affiliated Hospital of Fujian Medical University, Fuzhou, Fujian Province China; 7 Department of General Surgery, Sir Run Shaw Hospital, School of Medicine, Zhejiang University, Institute of Micro-invasive Surgery of Zhejiang University, Hangzhou, Zhejiang Province China; 8 Department of surgery, The University of Hong Kong-Shenzhen Hospital, Shenzhen, Guangdong Province China; 9 Department of Hepatobiliary Surgery, Weihai Central Hospital, Weihai, Shandong Province China; 10 Department of Minimally Invasive Surgery, Putuo Hospital Affiliated to Shanghai University of TCM, Shanghai, China

**Keywords:** Ventral hernia, Endoscopic sublay repair (ESR), Totally extraperitoneal sublay (TES), Transabdominal sublay (TAS), Total visceral sac separation (TVS) technique, Endoscopic transversus abdominis release (eTAR)

## Abstract

**Background:**

For ventral hernia, endoscopic sublay repair (ESR) may overcome the disadvantages of open sublay and laparoscopic intraperitoneal onlay mesh repair. This retrospective study presents the preliminary multicenter results of ESR from China. The feasibility, safety, and effectiveness of ESR were evaluated; its surgical points and indications were summarized.

**Methods:**

The study reviewed 156 ventral hernia patients planned to perform with ESR in ten hospitals between March 2016 and July 2019. Patient demographics, hernia characteristics, operative variables, and surgical results were recorded and analyzed.

**Results:**

ESR was performed successfully in 153 patients, 135 with totally extraperitoneal sublay (TES) and 18 with transabdominal sublay (TAS). In 19 patients, TES was performed with the total visceral sac separation (TVS) technique, in which the space separation is carried out along the peritoneum, avoiding damage to the aponeurotic structure. Endoscopic transversus abdominis release (eTAR) was required in 17.0% of patients, and only 18.3% of patients required permanent mesh fixation. The median operative time was 135 min. Most patients had mild pain and resume eating soon after operation. No severe intraoperative complications occurred. Bleeding in the extraperitoneal space occurred in two patients and was stopped by nonsurgical treatment. Seroma and chronic pain were observed in 5.23 and 3.07% of patients. One recurrence occurred after TAS repair for an umbilical hernia.

**Conclusion:**

ESR is feasible, safe, and effective for treating ventral hernias when surgeons get the relevant surgical skills, such as the technique of “partition breaking,” TVS, and eTAR. Small-to-medium ventral hernias are the major indications.

## Background

Ventral Hernia is a common surgical condition, which can be categorized into two types: primary ventral hernias and secondary ones. The former includes umbilical hernia, linear alba hernia, Spigelian hernia and lumbar hernia, while the latter refers to incisional hernia. Ventral hernia has a negative impact on quality of life and healthcare costs. The incidence of ventral hernia varies considerably among different reports. Take incisional hernia as an example, patients have a 2–20% risk of developing an incisional hernia after laparotomy [1]. Therefore, the surgical treatment of ventral hernia has always been the concern for general surgery. Currently, repairing the defect with placing prosthetics in different layers of the abdominal wall is the mainstream of surgery.

For ventral hernias, the effective repair planes include onlay, sublay, and intraperitoneal onlay mesh (IPOM). In sublay repair, a low recurrence rate is achieved because the mesh is placed in the retromuscular space behind the defect [2]. Compared with IPOM repair, neither the sublay repair procedure nor the mesh placement is in the abdominal cavity, reducing the risk of intraoperative bowel injury and postoperative mesh complications, such as bowel adhesion and fistula [3]. Compared with placing the mesh at the subcutaneous plane in onlay repair, in sublay repair, the mesh is far away from the skin, decreasing the risk of wound and mesh infection [2, 3]. Therefore, sublay is an ideal plane for ventral hernia repair in terms of effectiveness and safety. Sublay repair was initially performed as an open procedure. However, open sublay repair results in substantial trauma because of the extensive separation of the abdominal wall [2, 3]. Later, laparoscopic IPOM (Lap-IPOM) repair becomes one of the major procedures for ventral hernia repair because it makes the operation easy and causes less trauma; however, the intra-abdominal risks mentioned previously could not be overcome [3]. Additionally, the mesh fixation in IPOM repairmay cause postoperative pain [2, 3]. Thus, converting open sublay repair to endoscopic sublay repair (ESR) may result in the advantages of both the sublay repair and endoscopic techniques and seems to be a promising trend.

The first report of such a procedure was published by Miserez in 2002 [4]. Miserez’s paper described direct access to the retromuscular plane in a small cohort of 15 patients, and the procedure was referred to as “endoscopic totally preperitoneal” repair. However, no report of ESR with a totally extraperitoneal (TEP) approach can be found on PubMed in the following 15 years. The main reasons were the great technical difficulty of the procedure and the long operative time. However, this situation has changed in the last 2 years, as many articles about this emerging technique from different countries have been published [5–23].

Bittner [24] converted the “mini- or less-open sublay” (MILOS) technique to a transhernial endoscopic repair and named it the eMILOS technique. Belyansky [5] introduced his enhanced-view totally extraperitoneal (eTEP) procedure, which was originally used to treat complex inguinal hernias, to ventral hernia repair. Köhler and Baig operated on ventral hernias employing an extended totally extraperitoneal (also eTEP) approach [6, 7]. Similarly, several other scholars reported their cases with totally extraperitoneal sublay (TES) repair [8–11]. Prasad, Schroeder, and Masurkar reported cases in which they performed transabdominal sublay (TAS) repair based on the inguinal hernia transabdominal preperitoneal (TAPP) approach [16–18]. Moreover, robotic assistance was also introduced to ESR performed using either totally extraperitoneal [13–15] or transabdominal [19–23] approach.

In China, we began to perform ESR in the early 2010s, but the early practice was unintentional. For example, for a minor McBurney incisional hernia, extraperitoneal repair could be completed with an extended lateral space separation based on inguinal hernia TEP; for small suprapubic incisional hernias, the conventional transabdominal partial extraperitoneal (TAPE) repair could be replaced by opening the peritoneum higher on the cephalic side and placing the mesh entirely within the extraperitoneal space as that in TAPP. We once called them extended TEP and extended TAPP [25, 26]. Around 2015 to 2016, we began to realize the clinical value and the prospects of ESR, and since then, ESR has been performed by more Chinese surgeons.

Data from 156 planned ESR patients from ten Chinese hospitals were collected in this retrospective study, which is the second largest report of ESR after the report of Belyansky at the end of 2019 [15]. The primary endpoint of this study was to evaluate the feasibility, safety, and effectiveness of ESR, and the secondary endpoint was to summarize its associated surgical skills and indications. Furthermore, we also propose our theoretical opinion of ESR and discuss its prospects.

## Methods

### Patients

One hundred fifty-six patients with ventral hernia (including 12 cases combined with diastasis recti or only diastasis recti) who were recommended to undergo ESR in ten Chinese hospitals between March 2016 and July 2019 were included. Patient distribution was as follows: East Hospital affiliated to Tongji University (*n* = 30), Shanghai General Hospital (*n* = 22), Northeast International Hospital (*n* = 21), Linzi District People’s Hospital (*n* = 25), Shengjing Hospital (*n* = 15), The First Hospital affiliated to Fujian Medical University (*n* = 14), Sir Run Run Shaw Hospital (*n* = 10), The University of Hong Kong-Shenzhen Hospital (*n* = 8), Weihai Central Hospital (*n* = 6), and Putuo Hospital (*n* = 5). The qualifications of the surgeons included experience with > 500 inguinal hernia TEP or TAPP repairs, 50 Lap-IPOM and 50 open sublay repairs, and 5 ESRs.

### Surgical procedure and technical points

#### Trocar layout

**Fig. 1:**
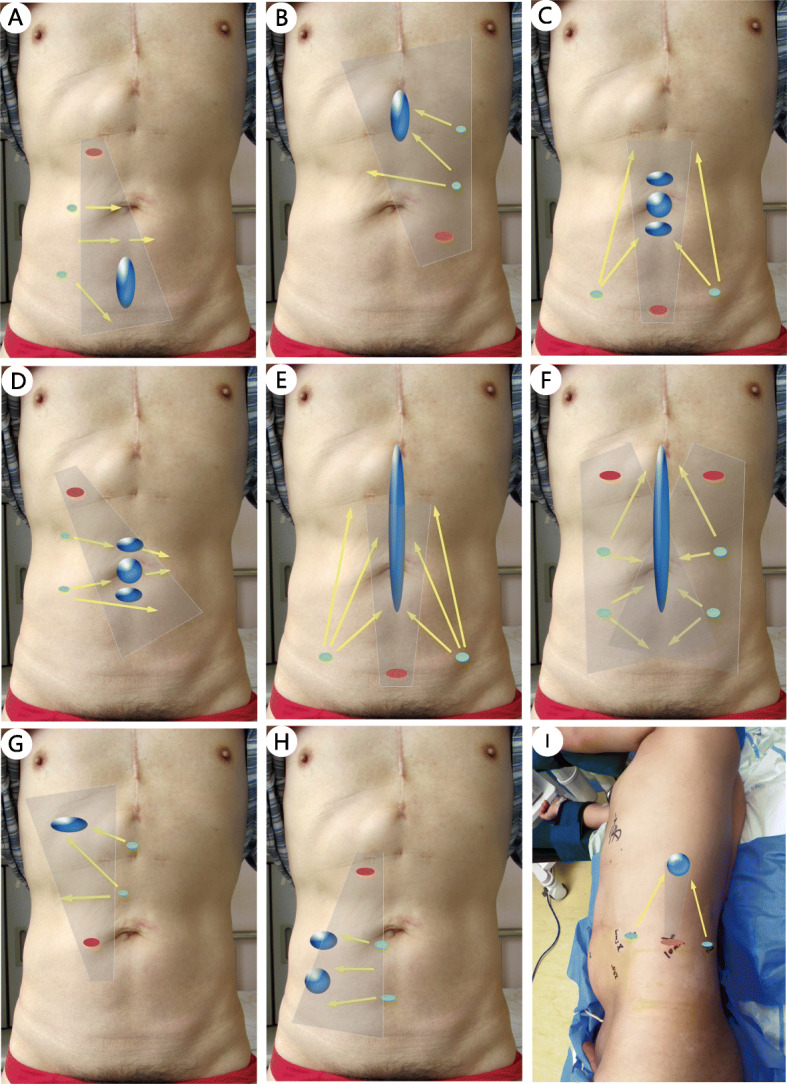
The typical trocar layout in TES for defects in different regions. **a**: M4 and/or M5; **b**: M1 and/or M2; **c–d**: M3; **e–f**: Long midline defect; **g**: L1; **h**: L4 and/or L5; **i**: L4; Gray shadow: Camera scope direction; Red dot: Camera trocar; Green dot: Surgeon trocars; *Region L, M is based on the incisional hernia classification of EHS [28]

**Fig. 2:**
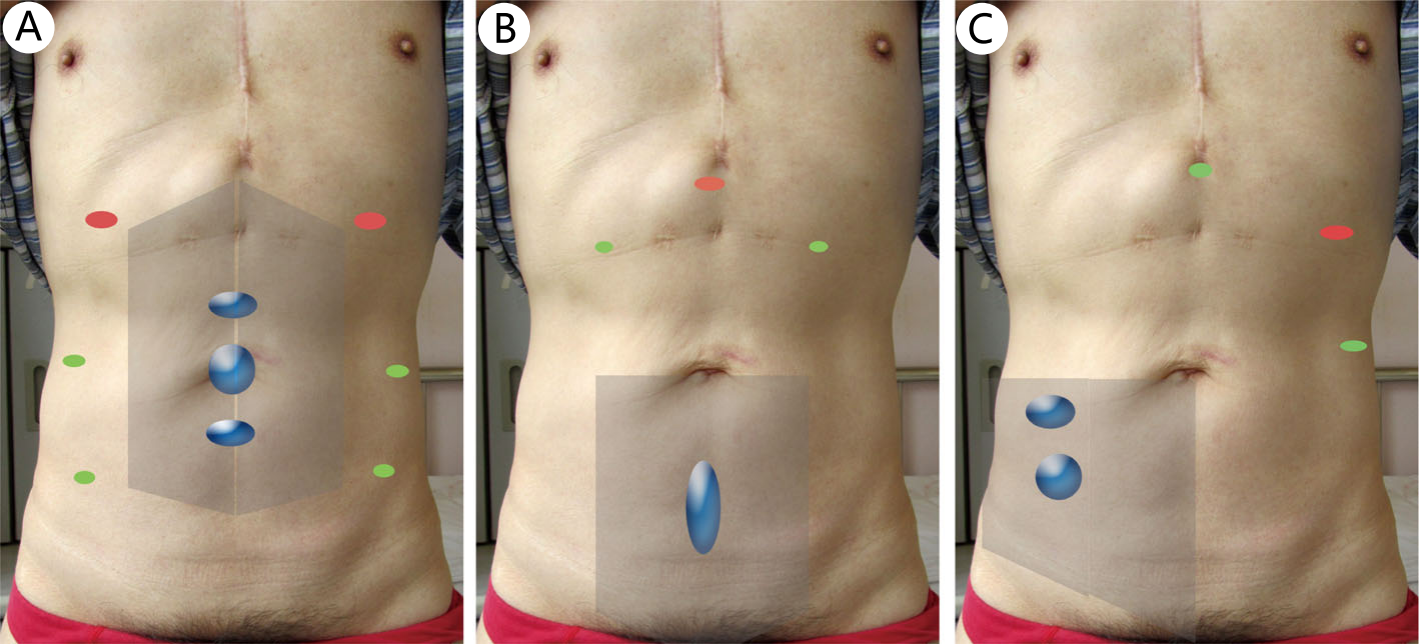
The typical trocar layout in TAS for defects in different regions. **a**: M2 and/or M3 and/or M4. **b**: M5 or (M5 and M4). **c**: L3 and/or L2; Shadow: Peritoneal flap that needs to be separated; Red dot: Camera trocar; Green dot: Surgeon trocars

In addition to following the basic principles of the trocar layout for routine endoscopic surgery, the defect location and the dissection plane should also be considered when positioning the trocars in ESR. Specifically, in the TES procedure, the camera and the operating trocar should first be placed where the sublay plane is easy to establish. After the establishment of part retromuscular space, more operating trocars are placed at appropriate locations. The trocar layout for different defect is relatively dynamic. In this article, we summarize the typical trocar layouts for various defect regions (Figs. 1 and 2). The camera trocar and the surgeon trocars can be switched when necessary.

#### The separation of the sublay plane

The sublay plane is also called the retromuscular space which includes the lateral space between the transversus abdominis and the peritoneum and the medial space between the rectus abdominis and the peritoneum.

**Fig. 3:**
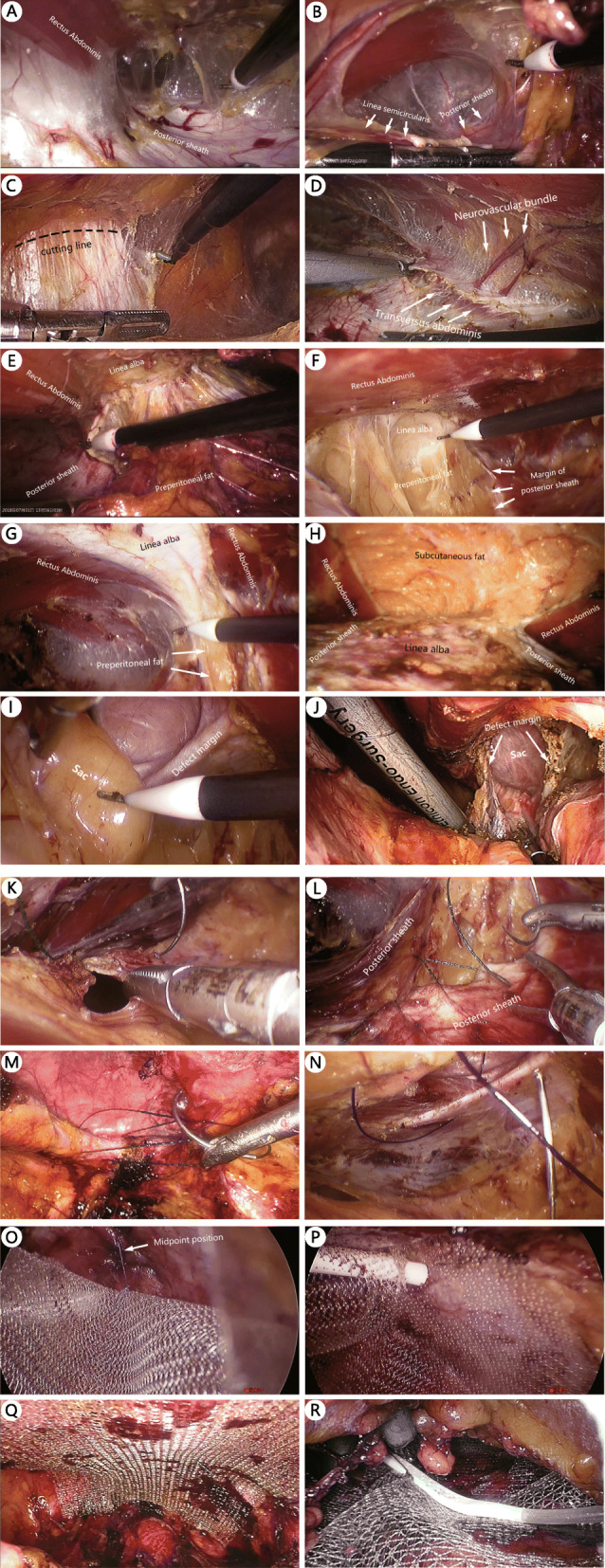
TES procedure. **a**: Separation in retro-rectus space. **b**: The entry of the retro-rectus space from above the linea semicircularis. Connecting Region I and Region II, below the umbilicus (**c**) and above the umbilicus (**d**). Connecting Region I of two sides, from caudal to cephalad (**e**) and from one side to another (**f**). **g**: The integrity of the linea alba and the anterior sheath is maintained. **h**: The integrity is broken

During ESR surgery, two planes where the posterior sheath exists can be dissected: the space anterior to the sheath and the space posterior to the sheath. The former space, also called the retro-rectus space, is the one most commonly used because it is easy to access and separate. Frequently during the TES procedure, after the retro-rectus space is accessed, a 10-mm trocar is placed in front of the posterior sheath (Figs. 3a) or directly in the extraperitoneal space above the pubis (Figs. 3b). Then, blunt dissection using the camera is performed to obtain more space. Afterward, several 5-mm trocars are placed in appropriate locations for further separation.

**Fig. 4:**
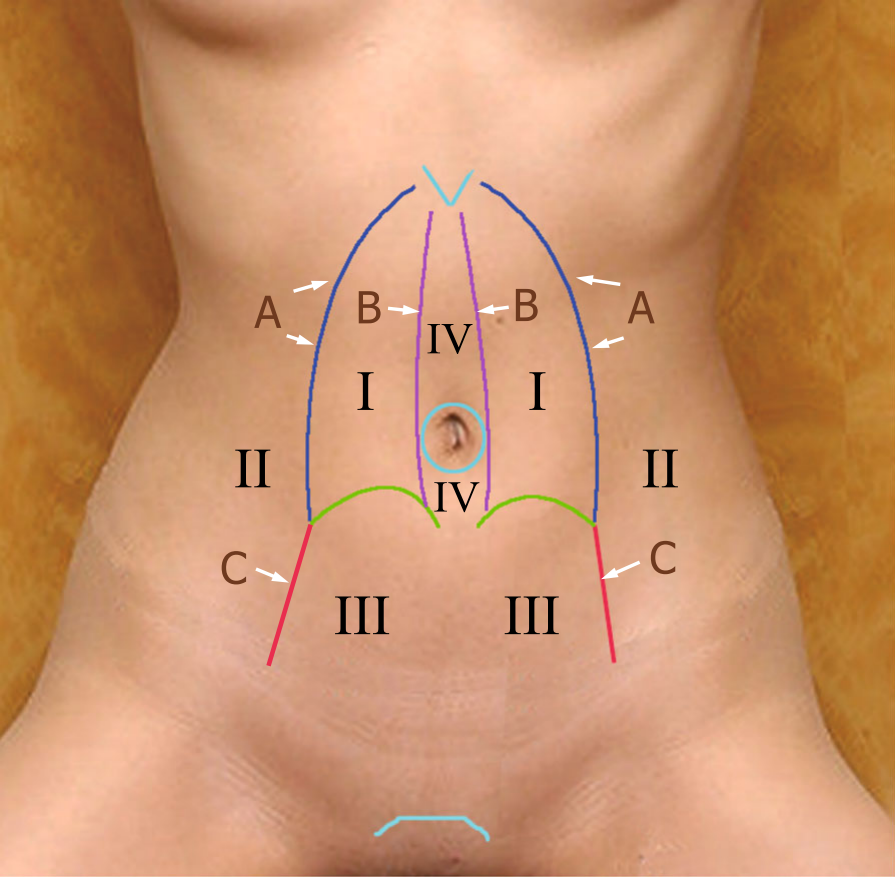
Partition of the abdominal wall. Region I: retro-rectus space above the linea semicircularis. Region II: Bogros space. Region III: Retzius space. Region IV: the space between the linea alba and the peritoneum, which is separated by the umbilicus. Line A: the outer edge of the posterior sheath inside of the neurovascular bundle and the transversus abdominis behind the sheath. Line B: the inner margin of the posterior sheath. Line C: the boundary between Retzius space and Bogros space

Anatomically, both the medial and lateral retromuscular spaces and the retro-rectus space on both sides are separated by several natural anatomic partitions and can be connected by breaking one or more of these partitions. So, referring to the partitions, we redivide the spaces of this plane into four regions (Fig. 4). We call the procedure of connecting the separated spaces by breaking partitions the “partition breaking” technique. First, the posterior sheath and the transversus abdominis behind are the partitions between Region I and the upper part of Region II “partition breaking” here is carried out by cutting Line A (Fig. 3c and d, this step is also called endoscopic transversus abdominis release (eTAR) [27]). Second, the skill of entering from Retzius space (Region III) to Bogros space (lower part of Region II) is precisely the same as that in TEP for inguinal hernia (breaking Line C). Third, “partition breaking” for connecting Region I on both sides is carried out by cutting off Line B and then dissecting Region IV to detach the extraperitoneal fat from the linea alba (Fig. 3e). The step performed from one side to another is called “crossover” (Fig. 3f). Consequently, the anterior sheath of both sides is still connected with the linea alba, which is crucial for preserving the integrity of the anterior abdominal wall (Fig. 3g and h). Additionally, the hernia ring and the umbilicus are both partitions too. The hernia sac reduction is easy (Fig. 3i) for primary ventral hernias but difficult for incisional hernias. The umbilicus or the sac is usually transected around the umbilicus or the hernia ring on the basis of the adhesions and scar in situ (Fig. 3j). Damaging the hernia contents must be avoided. After partition breaking, a large sublay plane crossing several retromuscular regions is obtained for subsequent operation and mesh deployment.

**Fig. 5:**
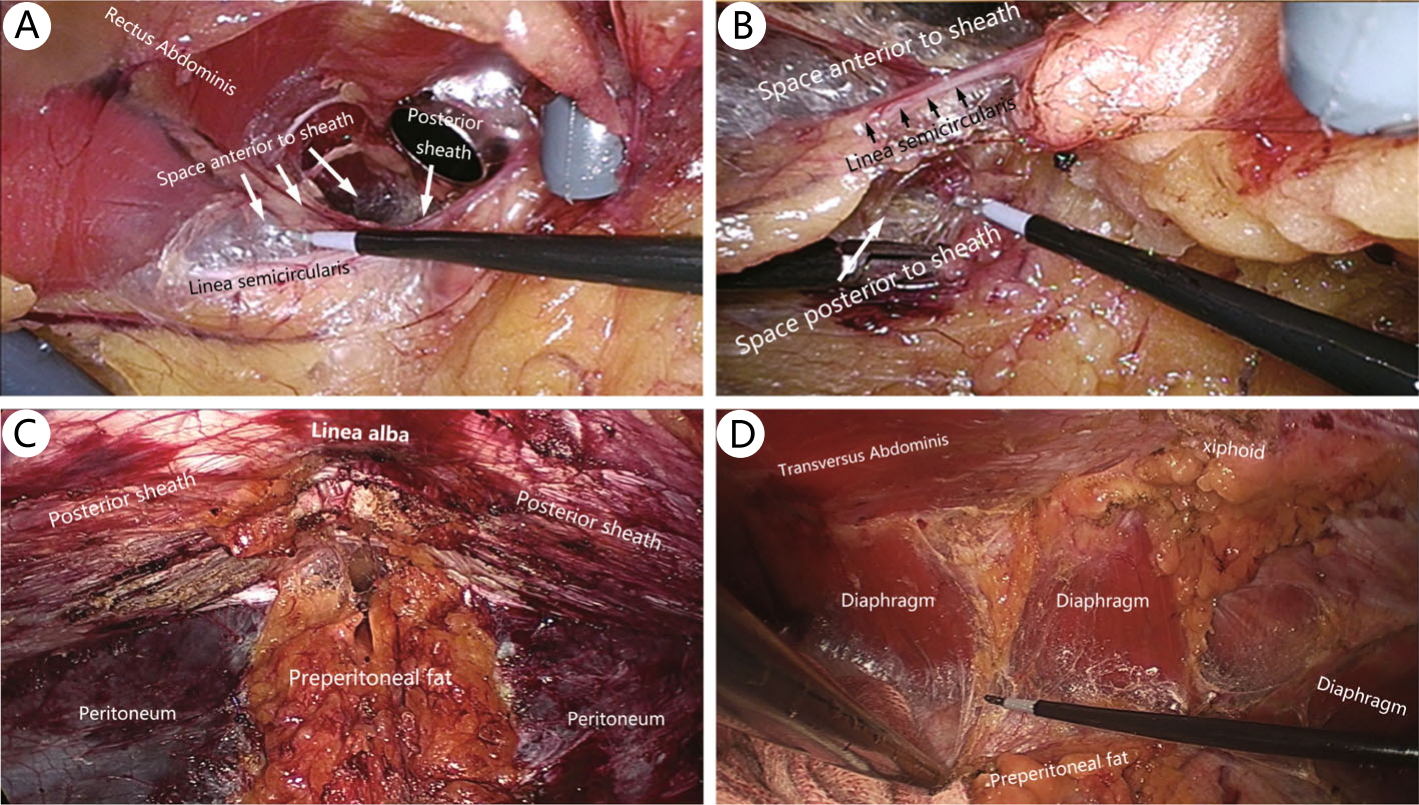
TVS procedure. **a**. Space anterior to the rectus sheath. **b**: Space posterior to the rectus sheath. **c**: Space below the umbilicus after separation. **d**: Space behind the xiphoid after separation

Another sublay plane where the posterior sheath exists is the space posterior to the sheath, this plane is also called preperitoneal layer (Fig. 5a and b). Here the peritoneum is quite thin and difficult to separate (Fig. 5b). The procedure is carried out by first creating a preperitoneal space above the pubis and then descending cephalad into the plane between the posterior sheath and the peritoneum, and further separating cranially and/or laterally (Fig. 5c and d). This space also directly connects with the lateral retromuscular space and then the retroperitoneum. Anatomically, the peritoneum is just like an eggshell membrane wrapping all abdominal viscera. The peritoneum on all sides, from bottom to top and from anterior to posterior, is connected and forms a whole visceral sac. Therefore, the technique of separating the whole visceral sac at this plane is called the total visceral sac separation (TVS), which was first proposed and implemented by Dr. Jiang HY [25]. It is a brand-new technique and will be illustrated in Discussion (Section 4).

**Fig. 6:**
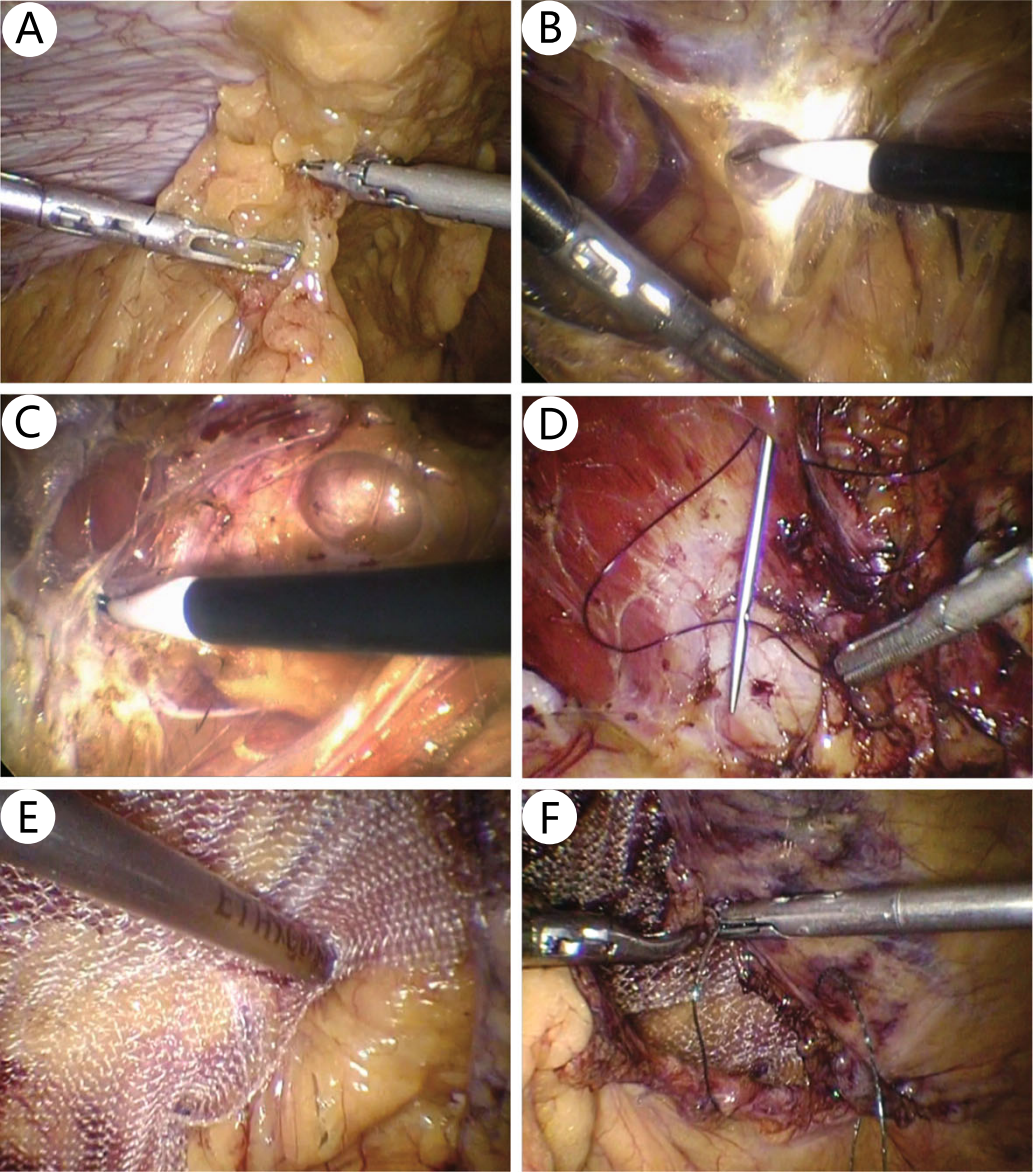
TAS procedure

The procedure for TAS is similar to that for the inguinal hernia TAPP. After entering the abdominal cavity, adhesiolysis is performed first (Fig. 6a). The peritoneum (mostly along with the posterior sheath) is then opened at least 5 cm away from the defect (Fig. 6b). The retromuscular space is further separated (Fig. 6c), and the range of separation includes the defect and at least 5 cm away from its margin.

#### Management of the defect

After space separation, the open peritoneum (Fig. 3k) or the peritoneum with the posterior sheath is closed with a continuous suture (Fig. 3l). The closure of the posterior sheath is not obligatory and is only implemented with low tension. Defect should be closed except few minor defects. The defect can be closed with either continuous suture with barbed or polyester thread (Fig. 3m) or interrupted transfascial suture with a suture passer (Figs. 3n and 6d).

#### Mesh deployment and fixation

After defect closure, a mesh is introduced into the separated space. The size of the mesh is related not only to the size of the defect but also to the range of the separation so that it can cover as much of the separated sublay plane as possible. At the maximum, when bilateral TAR was performed for a large midline defect, the mesh size would reach 30 × 35 cm; at the minimum, the mesh should cover not less than 5 cm away from the defect margin. The mesh can be placed without fixation (secured by just the abdominal pressure, sometimes with the central position; Fig. 3o) or with fewer points of fixation by glue (Fig. 3p), transfascial suture, or tackers. Self-adhesive mesh is also a good option (Fig. 3q). When a relatively small mesh is used, for example, if limited space is separated during TAS, the mesh should be fixed with multipoint transfascial sutures or tackers (Fig. 6e). A polypropylene mesh is used in most cases. Both normal-weight and light-weight mesh, including some partially absorbable meshes, are selected. If there are several points of small peritoneal damage, an anti-adhesion mesh is preferred.

#### Drainage and peritoneum closure

Closed drainage should be placed for patients with a large separated space, especially for the TES procedure (Fig. 3p). The peritoneal flap is closed with a continuous suture at the end of the TAS procedure (Fig. 6f).

### Data collection and follow-up

Surgical information, postoperative recovery, and short-term (within 1 month) and long-term (after 1 month) postoperative complications were carefully collected for further evaluation.

Each patient came to the clinic at 2 and 8 weeks, 6 months, and 1 year after surgery. After that, telephone interviews were conducted every half year, and patients with complaints visited the clinic to be checked for possible complications. Chronic pain was defined as sustained pain or discomfort that lasted > 3 months. The follow-up data collection was halted in October 2019, and the duration of follow-up ranged from 3 to 45 months (median 12 months; interquartile range [IQR] 5–21 months). One patient was lost to follow-up within 1 year, and five patients were lost to follow-up after 1 year.

### Statistical analysis

Qualitative data are expressed as percentage (%), and quantitative data as mean ± standard deviation or median (min–max) (IQR) concerning their distribution. Statistical assessments were conducted using Excel 2010.

## Results

The patient demographics and hernia characteristics are presented in Tables 1 and 2, respectively. Among 156 patients with planned ESR, 153 were operated on successfully. There were three conversion: one TAS was converted to TAPE because of insufficient peritoneal flap separation, one TAS was converted to Lap-IPOM because of severe damage to the peritoneum, and one TES was converted to an open procedure because of failure to create a retromuscular space.

**Table 1:** Patient demographics

	*N* = 156
Gender	
Male	73 (46.8%)
Female	83 (53.2%)
Age (year)	58.4 ± 14.8
BMI	25.1 ± 3.5
Operation history (time)	1 (0–5) (0–1)
ASA	
1	53 (34.0%)
2	95 (60.9%)
3	8 (5.13%)

**Table 2:** Hernia characteristics

	*N* = 156
Hernia type	
Primary ventral hernia	64 (41.0%)
Incisional hernia	92 (59.0%)
Defect region^a^	
Medial	107 (68.6%)
Lateral	43 (27.6%)
Crossing medial and lateral	6 (3.85%)
Defect width (W^a^)	
W1	104 (66.7%)
W2	50 (32.1%)
W3	2 (1.28%)
Defect size^b^ (cm^2^)	11.8 (1.8–70.7) (4.7–24.5)

^a^ Based on incisional hernia classification of the European Hernia Society (EHS) [28]

^b^Defect size (cm^2^) was calculated by area of an ellipse = π × (length/2 × width/2)

The operative variables and postoperative recovery are presented in Table 3. Other items to note are as follows. TVS and single-incision laparoscopic surgery (SILS) were all performed with the TES approach. Reduction of the hernia sac was achieved in 49 of 64 cases (76.6%) of primary ventral hernia and 35 of 89 cases (39.3%) of incisional hernia. Bilateral TAR was used to help close wide midline defects, and unilateral TAR was used to help reach the lateral defect from the medial region. The mesh was placed with permanent fixation in only 18.3% of patients.

**Table 3:** Operative variables and postoperative recovery

	*N* = 153
Procedure	
TES	135 (88.2%)
TAS	18 (11.8%)
TVS-TES	19 (12.4%)
SILS-TES	12 (7.84%)
Hernia sac management	
Reduction	84 (54.9%)
Transection	69 (45.1%)
TAR	
Without	127 (83.0%)
Unilateral	15 (9.80%)
Bilateral	11 (7.19%)
Defect closure	
No	7 (4.58%)
Suture (including barbed)	73 (47.7%)
Passer	69 (45.1%)
Passer + suture	4 (1.96%)
Mesh size (cm^2^)	150 (100–750) (135–225)
Mesh fixation	
No	63 (41.2%)
Temporary	62 (40.5%)
Self-adhesive mesh	33 (21.6%)
Glue	29 (19.0%)
Permanent	28 (18.3%)
Tack	22 (14.4%)
Transfascial	6 (3.92%)
Estimated blood loss (ml)	20 (10–100) (10–30)
Operative time (min)	135 (50–440) (115–180)
Drainage	89 (58.2%)
Drainage removal (days) ^a^	3 (1–10) (3–4)
Diet recovery (days)	1 (0.25–4) (0.25–2)
VAS^b^ 48 h	
Mild (1–3)	138(90.2%)
Moderate (4–6)	15 (9.8%)
Severe (7–10)	0 (0%)
Postoperative hospital stays (days)	3 (1–10) (2–4)

^a^ *n* = 91

^b^ visual analog scale (VAS) score (0–10)

The complications are presented in Table 4. During surgery, only one small bowel injury occurred, which was sutured without further event. Bleeding in the extraperitoneal space occurred in two patients after surgery. The drainage volume of the two patients was > 200 mL for the first four and six consecutive days after surgery, and the hemoglobin level dropped to < 80 g/L. The bleeding was stopped with nonsurgical treatment, but blood transfusion was required for one patient. Wound events occurred within 1 month in three patients; one patient who underwent TAS developed trocar site infection and was readmitted for secondary irrigation and debridement after > 1 month. One patient experienced ileus within 1 month postoperatively but recovered quickly after conservative treatment. No other severe short-term complications occurred. Seroma was observed in eight patients (5.23%) after > 1 month postoperatively; all seromas were absorbed with time or treated with needle aspiration. Other long-term complications included one recurrence after TAS repair of an umbilical hernia and five cases of chronic pain. The overall complication rate was 19.0%.

**Table 4:** Complications

	*N* = 153
Intraoperative complications	
Bleeding	0 (0.00%)
Visceral injury	1 (0.654%)
Short-term complications	
Wound events	3 (1.96%)
Seroma	4 (2.61%)
Hematoma	2 (1.31%)
Bleeding	2 (1.31%)
Edema	2 (1.31%)
Ileus	1 (0.65%)
Urinary tract infection	2 (1.31%)
Pneumonia	1 (0.65%)
Cardiovascular accident	0 (0.00%)
Deep vein thrombosis	0 (0.00%)
Long-term complications	
Seroma	8^a^ (5.23%)
Recurrence	1 (0.654%)
Trocar site hernia	0 (0.00%)
Wound infection	1 (0.654%)
Mesh infection	0 (0.00%)
Ileus	0 (0.00%)
Delayed intestinal fistulas	0 (0.00%)
Chronic pain	5 (3.07%)
Overall	29 (19.0%)

^a^ Including four cases whose seroma was not identified within 1 month after surgery

## Discussion

### Feasibility, safety, and effectiveness of ESR

Currently, this is the first comprehensive report of ESR from China; 153 of 156 included patients who had a planned ESR were operated on successfully, suggesting that this procedure is feasible for experienced hernia surgeons. Even if the ESR fails, there are still some fallbacks, such as Lap-TAPE, Lap-IPOM, and even open surgery. Although the operative time of ESR was much longer than Lap-IPOM because the dissection of a large sublay space under endoscopy was time-consuming, acceptable results in terms of intraoperative injury and postoperative recovery, as well as postoperative complications, were obtained. The median postoperative hospital stay of this series was 3 days, maybe a little longer than that of Lap-IPOM. The major cause is that drainage was placed in 58.2% of cases and most drainage was removed before discharge.

One crucial concern in the TES procedure is whether the separation of the hernia sac will damage the adherent bowel inside the sac. Our preventive measure was to open the sac around the hernia ring when it was difficult to separate, which allowed us to clearly see the intraperitoneal adhesion. Consequently, only one serosal injury of the small bowel occurred in this study.

In ESR, a large-size mesh is used with less fixation or even with no fixation when compared with that in Lap-IPOM. In Lap-IPOM, the defect is usually closed directly. During ESR, TAR is often needed for a large-to-medium midline defect, and it helps reduce the tension. Less fixation, defect closure with low tension, and no large incision as with the open sublay repair, these all contributed to mild postoperative pain. In TES procedure, the whole procedure and the mesh were totally in the extraperitoneal space, causing little interference with the abdominal cavity. Therefore, most patients resumed eating quickly.

The most serious postoperative complication observed was bleeding in the extraperitoneal space in two patients, both of whom had undergone TES repair. As the separated sublay space is large, it is difficult to control the bleeding once incomplete intraoperative hemostasis or postoperative bleeding occurs. This is a new complication that deserves our attention, and complete hemostasis should be achieved during surgery.

Up to the end of data collection, no mesh infection, delayed intestinal fistulas, or adhesive ileus was observed. However, one umbilical hernia with diastasis recti recurred 5 months after TAS surgery because of insufficient mesh coverage.

### The critical surgical skills

The first critical skill is the “partition breaking” technique. In ESR, we need to separate a large retromuscular space; however, the most commonly used sublay plane in the medial region, the retro-rectus space, is not connected with the lateral retromuscular space and the retro-rectus spaces of the other sides. Therefore, one or several anatomic partitions should be broken to satisfy the placement of a large mesh. The key points of the technique have been described in Section 2.

The second critical skill is TVS technique. For a large incisional hernia, it is worth breaking the partition. However, for a small ventral hernia, such as an umbilical hernia, cutting off the posterior sheath causes increased trauma and damages the integrity of the rectus sheath. It would be more appropriate to separate at another sublay plane, the plane between the posterior sheath and the peritoneum. This space not only directly connects with the lateral retromuscular space and then the retroperitoneum, but directly connects with the same space of other side. Equally important, along peritoneum, there is only some fascia structure. So, no aponeurosis or muscle needs to be cut off during separation. Anatomically, the peritoneum is just like an eggshell membrane wrapping all abdominal viscera and thus can be called as a visceral sac. The peritoneum from all sides (superior to inferior and anterior to posterior) connects and forms a whole visceral sac. The separation is implemented as if peeling an eggshell completely along its shell membrane. We named this technique of separating along the surface of the peritoneum, which potentially forms a whole visceral sac, as “total visceral sac separation (TVS)” technique [25]. Certainly, only in few hernia repairs, it needs to separate a wide range of visceral sac. However, it is potential to separate the visceral sac as large as possible at any region of abdominal wall. The separation of the whole visceral sac is difficult, especially at the part behind the posterior sheath, because the peritoneum there is very thin. Therefore, TVS is a challenging procedure, and its successful implementation requires more patience and time. In this study, 19 patients with TES repair from three hospitals were successfully operated on combining with the TVS technique. In another three patients with midline defects, the TVS technique failed because of peritoneal damage, and it were converted to routine TES procedure which operated at the retro-rectus plane. Additionally, TVS is especially suitable for primary ventral hernia repair, such as umbilical hernia, linear alba hernia and lumbar hernia.

Third, the trocar layout and procedure design are crucial for the successful implementation of ESR. Our layout and procedure are slightly different from those reported in the literature. Belyansky’s TES procedure [5] for midline defects usually employed a full-length crossover. We speculate that his procedure emphasized the separation of the entire retro-rectus space, facilitating the placement of a large mesh. Instead, we do not employ a full-length crossover. For example, for a small lower midline defect, if its margin is ≥5 cm away from the umbilicus, crossover is only implemented in the lower half of the abdomen, preserving the integrity of the posterior sheath above the umbilicus. Another probable cause for such difference is that the defect size in our study was much smaller than that in Belyansky’s study [5] (17.9 cm^2^ [calculated as an ellipse] versus 132.1 cm^2^ [calculated as a rectangle], respectively). In the TAS procedure, our procedure for lower midline defects was similar to that of Prasad [16] and Masurkar [18]. But for a large umbilical defect, Schroeder [17] and Masurkar [18] placed the trocars at the lateral region only on one side; the proximal posterior sheath and peritoneum together were then cut open. A whole peritoneal flap was prepared after the separation of the retro-rectus space of both sides. Instead, we preferred to raise two symmetrical flaps. Six trocars, three on each side, were placed at the lateral abdomen. The two flaps were raised by the instruments from the contralateral side and were sutured together on the midline after repair. This difference may be because robotic is used more frequently in TAS [19–23] than in TES [13–15]; however, the use of robotic is not widespread in hernia surgery in China. Therefore, suturing two flaps together on the midline is much easier than suturing one flap at a close range in routine endoscopic surgery. Moreover, the unilateral trocar layout was insufficient for implementing TAR on two sides.

### Terms

Different names, including *retromuscular*, *retro-rectus*, *Rives–Stoppa*, and *preperitoneal space*, have been used in the literature. These terms have nuanced differences resulting from the development history of Rives–Stoppa technology and the anatomical names of different abdominal wall regions [29]. However, in most cases, these terms are interchangeable and can be replaced by one name, *sublay*. Therefore, the different reports of similar procedures of placing the mesh at the sublay plane under laparoscopy can all be regarded as “laparoscopic sublay repair.” Because the totally extraperitoneal approach does not enter the abdominal cavity, we consider the term *endoscopic* to be broader and more appropriate than *laparoscopic*. Therefore, we call such operations as “endoscopic sublay repair (ESR)” for ventral hernia [25].

**Fig. 7:**
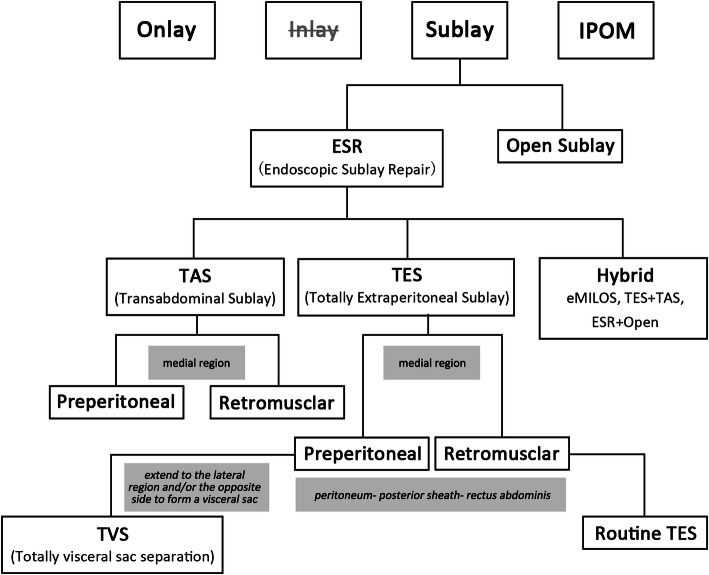
Procedures of sublay repair for ventral hernia

In terms of surgical approaches, there are also different expressions: *totally extraperitoneal*, *enhanced-view TEP*, *extended TEP*, *transperitoneal sublay*, *transabdominal retromuscular*, *TAPP*, and *extended TAPP*. Overall, the approaches of ESR for ventral hernias are the same as those for laparoscopic inguinal hernia repair (LIHR) and can be categorized as “totally extraperitoneal” and “transabdominal.” Adding “sublay” after “totally extraperitoneal” and “transabdominal,” we call the two procedures “totally extraperitoneal sublay” and “transabdominal sublay” (Fig. 7). Accordingly, we replace P/PP with S and abbreviate them as TES and TAS. Currently, the frequently used name for the extraperitoneal approach for ESR is *enhanced-view TEP* [5, 9]. This name was originally given to the procedure for complex LIHR in which the highly positioned camera provides an enhanced view, facilitating the subsequent dissection. However, when it was introduced to ESR for ventral hernia, it did not match the reality because no enhanced view seemed to be obtained. Therefore, we proposed a more generalized term, TES. Together with TAS, we recommend these two terms because they summarize and categorize the many reported procedures with various names. As TES and TAS correspond with those of LIHR, they are easy to remember and aid in understanding the procedures.

### Frequency of TES and TAS

In this study, about eight of nine cases were completed by TES. According to the experiences from LIHR, TAPP provides a larger working space, so the procedure is easier than TEP. However, the situation is different in ventral hernia. ESR requires a much larger separated sublay working space than LIHR does. In the TES procedure, the gas filling in the space between the peritoneum and the muscle depresses the peritoneum and helps separate a large working space. The instruments and operations in TES are all in the extraperitoneal space; however, the position of the instruments and the operations in TAS is split, making space separation in TES more direct and easier than that in TAS. Because the operation of TAS is toward the “ceiling,” the preparation and suturing of the peritoneal flap are difficult without robotic’s assistance. Above all, Chinese surgeons generally prefer TES over TAS.

### Limitations of the study

The follow-up was relatively short, and patients with no symptom were only followed up by telephone, so the recurrence rate may be underestimated. The patients were included from ten hospitals in different areas, so the indications, detailed procedure, and data evaluation were not definitely consistent. As a non-controlled retrospective study, we cannot conclude the differences of all variables between ESR and Lap-IPOM or open sublay repair. All of these deficiencies should be improved by standardized controlled studies in the future.

### Indications, contraindications, and prospect of ESR

For TES, small-to-medium ventral hernias (defect width < 6 cm) are the major indications. Without robotic’s assistance, the indication range for TAS is smaller than that for TES. TAS is suitable for small ventral hernias, especially for M5 and L3 defects. Contraindications include hernias with severe adhesion in the hernia sac, large-to-medium incisional hernias (defect width > 6 cm), or small defects combined with “loss of domain.”

Objectively, ESR is still a difficult operation. However, for small-to-medium ventral hernias, some disadvantages of Lap-IPOM and open sublay repair can be avoided if ESR is successfully implemented. With the generalization of ESR and the increase of experience, we believe that the indications for ESR will gradually extend. ESR, Lap-IPOM, and open sublay repair, together, will dominate the surgical treatment of ventral hernias for a long time in the future.

## Conclusion

ESR is a procedure that separates the retromuscular–extraperitoneal space and places a mesh at this sublay plane under endoscopy for the surgical treatment of ventral hernia. From the results of this study, we consider ESR to be feasible, safe, and effective for the treatment of ventral hernias when the surgeons have an in-depth anatomic knowledge of the abdominal wall and relevant surgical skills. Small-to-medium incisional hernias and primary ventral hernias are the major indications for ESR.

## Supplementary information

**Additional file 1.**

## Data Availability

All results generated or analyzed during this study are included in the article, and the original collected data form is attached as a supplement.

## Abbreviations

ESR: Endoscopic sublay repair
TES: Totally extraperitoneal sublay
TAS: Transabdominal sublay
TVS: Total visceral sac separation
TAR: Transversus abdominis release
eTAR: Endoscopic transversus abdominis release
IPOM: Intraperitoneal onlay mesh
Lap-IPOM: Laparoscopic intraperitoneal onlay mesh
TEP: Totally extraperitoneal
eTEP: Enhanced-view totally extraperitoneal
TAPP: Transabdominal preperitoneal
TAPE: Transabdominal partial extraperitoneal
IQR: Interquartile range
